# The Association Between Cognitive Impairment and Subsequent Falls Among Older Adults: Evidence From the China Health and Retirement Longitudinal Study

**DOI:** 10.3389/fpubh.2022.900315

**Published:** 2022-06-15

**Authors:** Rong Zhou, Jiayu Li, Meiling Chen

**Affiliations:** ^1^The Second School of Clinical Medicine, Zhejiang Chinese Medical University, Hangzhou, China; ^2^School of Humanities and Management, Zhejiang Chinese Medical University, Hangzhou, China

**Keywords:** falls, cognitive domains, cognitive function, older adults, subgroup analysis

## Abstract

**Introduction:**

Previous studies have suggested that cognitive impairment is associated with falls in older adults. However, the consistency of results among different subgroups defined by multiple characteristics of the elderly has not yet been tested. Additionally, results are inconsistent regarding the effects of different cognitive domains on falls. Therefore, this study sought to use representative data from a nationwide study to better understand the longitudinal association between cognitive impairment and subsequent falls in older adults.

**Methods:**

The current study was conducted based on the China Health and Retirement Longitudinal Study (CHARLS) data of respondents aged ≥60 years in 2015 and the fall data in 2018. The respondents were divided into subgroups according to different demographic characteristics. Multiple logistic regression analysis was conducted to adjust for various confounding factors and evaluate the association between cognitive impairment and falls.

**Results:**

Of the 5,110 respondents included in this study, 1,093 (21.39%) had falls within the last 2 years. A significant association was found between cognitive impairment and subsequent falls (OR = 0.97, 95% CI 0.95–0.99, *P* = 0.001) after adjusting for all of the covariates related to falls. Analysis of different cognitive domains showed that orientation (OR = 0.94, 95% CI 0.90–0.99, *P* = 0.013) and memory (OR = 0.93, 95% CI 0.90–0.97, *P* = 0.001) were significantly associated with falls. In subgroup analysis, the ORs of people aged 60–74 years, male, with lower education level were 0.97 (95% CI 0.95–0.99, *P* = 0.008), 0.96 (95% CI 0.93–0.98, *P* = 0.001), and 0.97 (95% CI 0.95–0.99, *P* = 0.001), respectively, suggesting that the associations were also statistically significant in these subgroups. There was also a significant association both in urban (OR = 0.97, 95% CI 0.95–0.99, *P* = 0.001) and in rural residents (OR = 0.97, 95% CI 0.95–0.99, *P* = 0.003).

**Conclusions:**

Our results suggest that the associations between cognition and falls vary depending on the different demographic characteristics of older adults. These findings may be useful for designing more accurate identification and intervention for the fall risk for specific high-risk groups.

## Introduction

Falls are a serious public health issue for the aging population. According to the World Health Organization (WHO) estimates, one-third of community-dwelling people ≥65 years of age experience falls every year, and nearly half of them sustain repeated falls (1, 2). Falls seriously harm the physical and mental health of older adults, bringing a heavy burden on the family and society as well (3, 4). Therefore, identification of risk factors related to falls can help in formulating effective prevention strategies to improve the health care system in an aging society.

Falls are caused by the interaction of internal and external risk factors (5, 6), which have been extensively investigated in many cross-sectional and longitudinal studies. Intrinsic risk factors include demographic characteristics (such as age, gender, and education level), lifestyle behaviors (such as smoking, alcohol abuse, sleep duration, and sleep disturbance) (7–9), health status (such as cognition, physical function, and chronic diseases), and history of falls. Common external risk factors include medication, environmental hazards, and hazardous activities (10).

Many studies have revealed that cognitive impairment is a risk factor for falls among older adults (10, 11). However, the elderly are not a homogeneous group, and such associations may vary depending on the characteristics of older adults. It has been found that cognitive function has a value in predicting falls among older adults when they are regarded as a unique group (12, 13); however, it might have a greater value in subgroups with certain characteristics, but this aspect has been barely explored in previous studies. Subgroup analyses may provide valuable information as to whether the study conclusions vary depending on different demographic characteristics. Thus, to obtain more accurate evidence for fall prevention in the elderly, it is meaningful to carry out subgroup analysis from multiple dimensions according to the characteristics of the elderly.

In addition, cognitive function encompasses different domains such as memory, visuospatial ability, orientation, calculation, execution, and comprehension. Cognitive impairment refers to the decline of one or more than one of the above cognitive functions. Dementia is diagnosed when the cognitive function decline involves two or more of the above cognitive domains and affects one's daily or social abilities (14, 15). Previous studies have documented that measurements of the main cognitive domains can be useful in discriminating the preclinical stage of Alzheimer's disease (15, 16), which may also help to identify individuals at high risk of falls. However, studies on cognitive domains and falls have yielded mixed results (13, 17–19).

Therefore, this study aimed to use representative data from a nationwide study to better understand the association between cognitive impairment and subsequent falls in older adults after adjusting for various factors, adding to evidence on impairment of the main cognitive domains as a predictor of falls. Subgroup analyses were conducted to testify whether the significance of this association still exists in subgroups defined by different demographic characteristics of the elderly, so as to design targeted prevention and intervention measures for selected high-risk populations.

## Materials and Methods

### Participants

The China Health and Retirement Longitudinal Study (CHARLS) is a biennial survey that collects a set of high-quality longitudinal data representing individuals and families of people aged ≥45 years in China. The baseline data were launched in 2011 and tracked every 2–3 years. The questionnaire of CHARLS covers many aspects of health-related information, including cognition and self-reported falls. The CHARLS data are available publicly on the China Health and Retirement Longitudinal Study website under the auspices of the National Development Research Institute of Peking University.

In this study, data on demographic characteristics, lifestyle behaviors, health status, and cognition were acquired from the 2015 database of CHARLS as the baseline data. Data on falls were acquired from the tracking data in 2018, and were used as the follow-up data.

**Figure 1:**
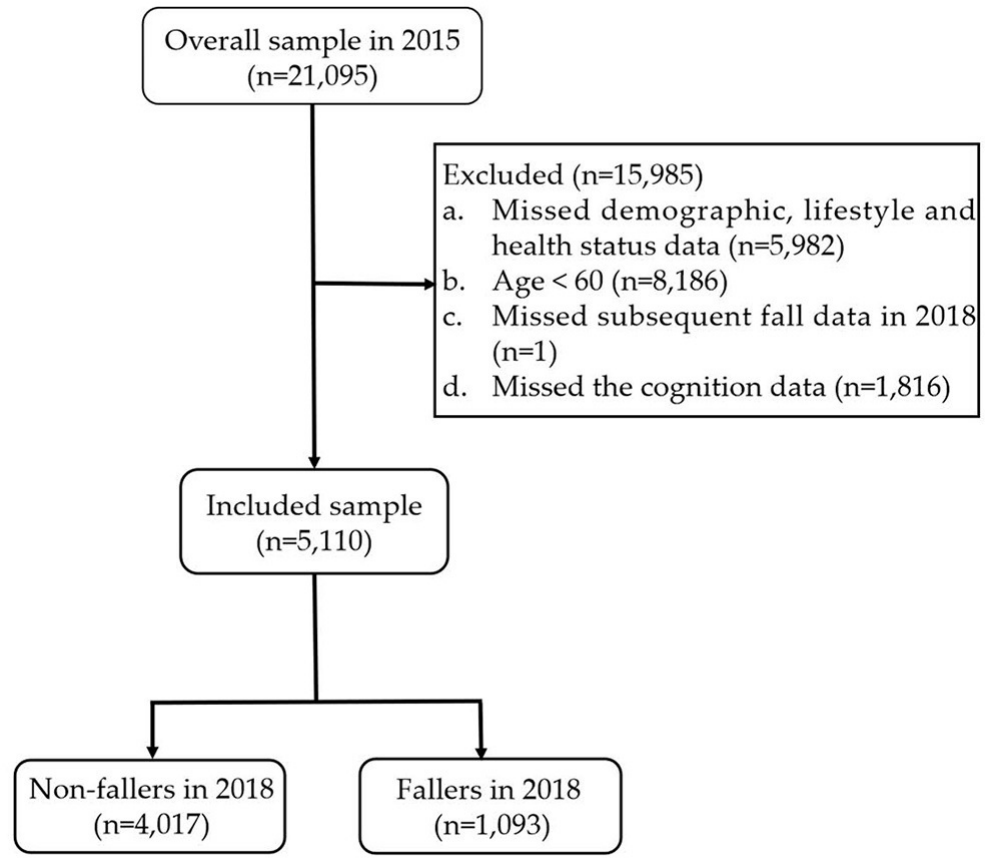
Participants included in the analysis.

Participants aged ≥60 years were selected for inclusion in the study. The exclusion criteria were as follows: (1) missing data on demographic characteristics, lifestyle behaviors, and health status in 2015; (2) age <60 years; (3) missing data on falls in 2018; (4) missing data on cognition in 2015. According to these exclusion criteria, we discarded 15,985 individuals from the overall sample in the 2015 database. Ultimately, a total of 5,110 individuals were included in this study. The detailed exclusion process is shown in Figure 1.

### Fall

Fall was an important outcome index of this study. In the CHARLS survey, participants were asked to answer “yes” or “no” to the question “Have you fallen in the last 2 years?”

### Cognition

The assessment of cognitive function in the CHARLS questionnaire includes telephone interviews for cognitive status-10 (TICS-10), visuospatial ability test, and episodic memory capacity. TICS-10 mainly measures the abilities of orientation and calculation, with a total score of 10 points. Respondents were required to answer the year, season, date, day of the week, and month in which the survey was conducted; one point was given for each correct answer. Next, the respondents needed to perform five serial subtractions of 7 without reminding, one point will be given for each correct calculation. And if there were two consecutive calculation errors, no points will be given for subsequent answers. Regarding the visuospatial ability, the respondents were asked to draw pictures displayed by the interviewer, and the correctly drawn picture was worth 1 point. Episodic memory capacity was tested by phrase recall; specifically, the respondents were asked to recall 10 phrases provided by the interviewer, with a total score of 10. The total score of the above tests was regarded as the overall cognitive function score, with a maximum score of 21. Older adults with higher scores were considered to have a better cognitive function (20, 21).

### Controlled Variables

In this study, the controlled variables were as follows: (1) demographic characteristics: age, gender, address, marital status, and education level obtained through face-to-face interviews; (2) lifestyle and behavior: smoking, drinking, and sleep status; (3) health status: Body mass index (BMI) was obtained by calculating the height and weight data measured by CHARLS physical examination (divided into four categories: underweight, ≤18.4 kg/m^2^; normal weight, 18.5 kg/m^2^ ≤BMI <24 kg/m^2^; overweight, 24 kg/m^2^ ≤BMI <28 kg/m^2^; and obese, ≥28 kg/m^2^). Moreover, the CHARLS questionnaire investigates 14 chronic diseases, including hypertension, dyslipidemia, diabetes, chronic lung diseases, liver disease, heart attack, stroke, kidney disease, digestive disease, emotional, nervous, or psychiatric problems, memory-related disease, arthritis, and asthma. The participants were asked to answer “yes” or “no” to the question “Have you been diagnosed with conditions listed below by a doctor?” Disability variables included physical disabilities, brain damage/intellectual disability, vision problems, hearing problems, and a speech impediment. History of falls was obtained by asking “Have you fallen since your last visit?” (4) Physical examination and functional test: Depressive symptoms were measured by the CES-D scale, with a maximum total score of 30. The critical value of ≥10 was used to determine the presence of significant depressive symptoms in this study (22, 23). The balance test required each participant to stand with the heel of one foot in front of the other, touching the toes of the other foot for about 30/60 s (30 s for individuals aged ≥70 years; 60 s for individuals <70 years). The result was recorded as “pass” or “fail”.

### Statistical Analyses

IBM SPSS Statistics v26 was used for statistical analyses. The included subjects were divided into two groups, including the non-fallers group and the fallers group. Categorical variables were expressed as absolute numbers and proportions (%) of the total. Continuous variables were tested for normal distribution by the Kolmogorov-Smirnov test. Non-normally distributed variables were expressed as the median (IQR) and normally distributed continuous variables were expressed as mean ± SD. The Student's *t*-test for continuous variables and the chi-square test for categorical variables were used for comparison of the characteristics between the two groups.

The association between cognitive function (the score of cognition as a continuous variable) and falls was evaluated by binary logistic regression models, controlled for the confounding factors. The unadjusted model was a crude model without adjustment for any of the covariates. Model 1 was adjusted for covariates including age and gender. Model 2 was adjusted for covariates including age, gender, marital status, smoking, physical disabilities, intellectual disability, night sleep duration, depression, chronic diseases, and history of falls. In subgroup analysis, the participants were divided into subgroups based on demographic characteristics including age, gender, location, and education level. Binary logistic regression was conducted in each of the subgroups after adjusting for all of the covariates listed above (model 3). The odds ratios (ORs) and 95% confidence intervals (CIs) were calculated. A two-sided *P*-value < 0.05 was considered statistically significant in the current study.

## Results

### Comparison of General Characteristics Between the Non-fallers Group and the Fallers Group

Table 1 provides an overview of the demographic characteristics of the included study subjects. Among the 5,110 subjects included in the study, a total of 1,093 (21.39%) experienced falls within the last 2 years. In terms of demographic characteristics, the incidence of falls was higher in women than in men (25.99 vs. 17.57%), and gender significantly correlated with falls (χ^2^ = 53.39, *P* < 0.001). Increased age was significantly related to a higher possibility of falls (χ^2^ = 4.98, *P* < 0.001). Moreover, falls tended to occur in older adults with lower educational level (χ^2^ = 7.18, *P* = 0.028) and unfavorable marital status (χ^2^ = 17.19, *P* < 0.001).

**Table 1:** Comparison of demographic characteristics between the non-fallers group and the fallers group (*n* = 5,110).

	**Total**	**Fall**		**χ^2^/t**	***P*-value**
		**Yes**	**No**		
Age	67.26 ± 5.92	68.05 ± 6.23	67.05 ± 5.82	4.98	<0.001***
**Gender**, ***n*** **(%)**				53.39	<0.001***
Male	2,794 (54.68)	491 (17.57)	2,303 (82.43)		
Female	2,316 (45.32)	602 (25.99)	1,714 (74.01)		
**Location**, ***n*** **(%)**				0.26	0.879
Urban areas	761 (14.89)	162 (21.29)	599 (78.71)		
Rural areas	3,672 (71.86)	791 (21.54)	2,881 (78.46)		
Other	677 (13.25)	140 (20.68)	537 (79.32)		
**Marital status**, ***n*** **(%)**				17.19	<0.001***
Married with spouse present	4,132 (80.86)	836 (20.23)	3,296 (79.77)		
Other	978 (19.14)	257 (26.28)	721 (73.72)		
**Education**, ***n*** **(%)**				7.18	0.028*
Primary school or below	3,841 (75.17)	854 (22.23)	2,987 (77.77)		
Middle school	842 (16.48)	164 (19.48)	678 (80.52)		
High school or above	427 (8.36)	75 (17.56)	352 (82.44)*		

* *P < 0.05;*

*** *P < 0.001*.

As shown in Table 2, there were statistically significant differences in smoking status (χ^2^ = 12.12, *P* < 0.001), physical disabilities (χ^2^ = 41.95, *P* < 0.001), intellectual disability (χ^2^ = 19.62, *P* < 0.001), vision problems (χ^2^ = 11.23, *P* = 0.001), hearing problems (χ^2^ = 10.43, *P* = 0.001), night sleep duration (χ^2^ = 49.58, *P* < 0.001), nap duration (χ^2^ = 14.95, *P* = 0.002), balance (χ^2^ = 11.63, *P* = 0.001), chronic diseases (χ^2^ = 15.37, *P* < 0.001), depression (χ^2^ = 56.35, *P* < 0.001), and history of falls (χ^2^ = 261.42, *P* < 0.001) between fallers and non-fallers. However, there were no significant differences in drinking behavior, speech impediment, and BMI between fallers and non-fallers (*P* > 0.05). In addition, there was significant association between cognition and falls (*t* = −6.73, *P* < 0.001).

**Table 2:** Comparison of controlled variables between the non-fallers group and the fallers group (*n* = 5,110).

**Variables**	**Total**	**Fall**		**χ^2^/t**	***P*-value**
		**Yes**	**No**		
**Smoking**, ***n*** **(%)**				12.12	<0.001***
Yes	1,842 (36.05)	345 (18.73)	1,497 (81.27)		
No	3,268 (63.95)	748 (22.89)	2,520 (77.11)		
**Drinking behavior**, ***n*** **(%)**				1.74	0.187
Yes	1,840 (36.01)	375 (20.38)	1,465 (79.62)		
No	3,270 (63.99)	718 (21.96)	2,552 (78.04)		
**Physical disabilities**, ***n*** **(%)**				41.95	<0.001***
Yes	409 (8.00)	139 (33.99)	270 (66.01)		
No	4,701 (92.00)	954 (20.29)	3,747 (79.71)		
**Intellectual disability**, ***n*** **(%)**				19.62	<0.001***
Yes	363 (7.10)	111 (30.58)	252 (69.42)		
No	4,747 (92.90)	982 (20.69)	3,765 (79.31)		
**Vision problems**, ***n*** **(%)**				11.23	0.001**
Yes	775 (15.17)	201 (25.94)	574 (74.06)		
No	4,335 (84.83)	892 (20.58)	3,443(79.42)		
**Hearing problems**, ***n*** **(%)**				10.43	0.001**
Yes	1,108 (21.68)	276 (24.91)	832 (75.09)		
No	4,002 (78.32)	817 (20.41)	3,185 (79.59)		
**Speech impediment**, ***n*** **(%)**				1.53	0.217
Yes	41 (0.80)	12 (29.27)	29 (70.73)		
No	5,069 (99.20)	1,081 (21.33)	3,988 (78.67)		
**Night sleep duration (in hours)**, ***n*** **(%)**				49.58	<0.001***
<6	1,608 (31.47)	437 (27.18)	1,171 (72.82)		
6–8	3,022 (59.14)	552 (18.27)	2,470 (81.73)		
>8	480 (9.39)	104 (21.67)	376 (78.33)		
**Nap duration (in minutes)**, ***n*** **(%)**				14.95	0.002**
0	1,971 (38.57)	457 (23.19)	1,514 (76.81)		
<30	354 (6.93)	86 (24.29)	268 (75.71)		
30–60	1,725 (33.76)	364 (21.10)	1,361 (78.90)		
>60	1,060 (20.74)	186 (17.55)	874 (82.45)		
**Balance test**, ***n*** **(%)**				11.63	0.001**
Fail	128 (2.50)	43 (33.59)	85 (66.41)		
Pass	4,982 (97.50)	1,050 (21.08)	3,932 (78.92)		
**BMI (kg/m**^**2**^**)**, ***n*** **(%)**				4.71	0.194
≤18.4	325 (6.36)	85 (26.15)	240 (73.85)		
18.5–23.9	2,616 (51.19)	549 (20.99)	2,067 (79.01)		
24–27.9	1,599 (31.29)	339 (21.20)	1,260 (78.80)		
≥28	570 (11.15)	120 (21.05)	450 (78.95)		
**Number of chronic diseases**, ***n*** **(%)**				15.37	<0.001***
0	1,145 (22.41)	197 (17.21)	948 (82.79)		
≥1	3,965 (77.59)	896 (22.60)	3,069 (77.40)		
**CES-D score**, ***n*** **(%)**				56.35	<0.001***
0–9	3,151 (61.66)	567 (17.99)	2,584 (82.01)		
10–30	1,959 (38.34)	526 (26.85)	1,433 (73.15)		
**History of falls**, ***n*** **(%)**				261.42	<0.001***
Yes	948 (18.55)	387 (40.82)	561 (59.18)		
No	4,162 (81.45)	706 (16.96)	3,456 (83.04)		
Cognition	11.62 ± 3.76	10.94 ± 3.95	11.80 ± 3.68	−6.73	<0.001***

** *P < 0.01;*

*** *P < 0.001*.

### Comparison of Cognitive Domains of the Non-fallers Group and the Fallers Group

The assessment of cognitive function in the CHARLS questionnaire included four main domains of cognition (orientation, calculation, visuospatial ability, and memory). Further analysis of the cognitive domains showed that orientation (*t* = 5.13, *P* < 0.001), memory (*t* = 4.92, *P* < 0.001), visuospatial ability (χ^2^ = 11.54, *P* = 0.001), and calculation (*t* = 5.05, *P* < 0.001) were significantly associated with the incidence of falls in older adults (Table 3).

**Table 3:** Comparison of cognitive domains between the non-fallers group and the fallers group (*n* = 5,110).

	**Total**	**Fall**		**χ^2^/t**	***P*-value**
		**Yes**	**No**		
Orientation	3.76 ± 1.39	3.57 ± 1.47	3.81 ± 1.37	5.13	<0.001***
Memory	3.65 ± 1.76	3.42 ± 1.81	3.72 ± 1.74	4.92	<0.001***
Calculation	3.59 ± 1.54	3.38 ± 1.63	3.65 ± 1.51	5.05	<0.001***
**Visuospatial ability**, ***n*** **(%)**				11.54	0.001**
0	1,957 (38.30)	467 (23.86)	1,490 (76.14)		
1	3,153 (61.70)	626 (19.85)	2,527 (80.15)		

** *P < 0.01;*

*** *P < 0.001*.

### Logistic Regression Analysis of Cognition and Subsequent Falls

As shown in Table 4, older adults with better cognitive function had a lower risk of falls (OR = 0.90, 95% CI 0.89–0.90, *P* < 0.001) in the unadjusted model. For each additional point in cognitive function, the risk of falls can be reduced by 10%. The association remained significant after adjusting for all of the covariates, including age, gender, marital status, smoking, physical disabilities, intellectual disability, night sleep duration, depression, chronic diseases, and history of falls in model 2 (adjusted OR = 0.97, 95% CI 0.95–0.99, *P* = 0.001).

**Table 4:** Logistic regression analysis of cognitive function and subsequent falls (*n* = 5,110).

	**Unadjusted model**	**Model 1**		**Model 2**		
	**OR (95% CI)**	***P***	**OR (95% CI)**	***P***	**OR (95% CI)**	***P***
Gender (ref: male)	–	–	1.43 (1.25–1.63)	<0.001***	1.43 (1.20–1.69)	<0.001***
Age	–	–	0.99 (0.98–0.99)	<0.001***	1.01 (1.00–1.01)	0.003**
Martial Status (ref: married with spouse present)	–	–	–	–	1.18 (0.99–1.40)	0.064
Smoking (ref: yes)	–	–	–	–	0.95 (0.79–1.13)	0.537
Physical disabilities (ref: yes)	–	–	–	–	0.55 (0.44–0.69)	<0.001***
Intellectual disability (ref: yes)	–	–	–	–	0.70 (0.55–0.89)	0.004**
Night sleep duration (in hours) (ref: <6)	–	–	–	–		0.002**
6–8	–	–	–	–	0.76 (0.66–0.89)	0.001**
>8	–	–	–	–	0.88 (0.68–1.13)	0.306
CES-D score (ref: 0–9)	–	–	–	–	1.26 (1.09–1.45)	0.002**
Chronic diseases (ref: 0)	–	–	–	–	1.09 (0.91–1.30)	0.35
History of falls (ref: no)	–	–	–	–	0.34 (0.29–0.40)	<0.001***
Cognition	0.90 (0.89–0.90)	<0.001***	0.94 (0.92–0.95)	<0.001***	0.97 (0.95–0.99)	0.001**

** *P < 0.01;*

*** *P < 0.001. OR, odds ratio; 95% CI, 95% confidence interval. Unadjusted model: without adjustment for any covariates. Model 1 was adjusted for covariates including age and gender. Model 2 was adjusted for covariates including age, gender, marital status, smoking, physical disabilities, intellectual disability, night sleep duration, depression, chronic diseases, and history of falls*.

Table 5 shows the results of logistic regression analysis of the associations between the main cognitive domains and subsequent falls. The OR values of orientation, memory, calculation, and visuospatial ability were 0.72 (95% CI 0.71–0.73, *P* < 0.001), 0.72 (95% CI 0.71–0.74, *P* < 0.001), 0.72 (95% CI 0.70–0.73, *P* < 0.001), and 0.25 (95% CI 0.23–0.27, *P* < 0.001), respectively, in the unadjusted model. After adjusting for age and gender (model 1), these associations remained significant. Model 2 showed that orientation (OR = 0.94, 95% CI 0.90–0.99, *P* = 0.013) and memory (OR = 0.93, 95% CI 0.90–0.97, *P* = 0.001) were two independent predictors of falls after adjusting for all of the covariates listed above.

**Table 5:** Logistic regression analysis of the four cognitive domains and subsequent falls (*n* = 5,110).

	**Unadjusted model**	**Model 1**		**Model 2**		
	**OR (95% CI)**	***P***	**OR (95% CI)**	***P***	**OR (95% CI)**	***P***
Orientation	0.72 (0.71–0.73)	<0.001***	0.87 (0.83–0.91)	<0.001***	0.94 (0.90–0.99)	0.013*
Memory	0.72 (0.71–0.74)	<0.001***	0.88 (0.85–0.91)	<0.001***	0.93 (0.90–0.97)	0.001**
Calculation	0.72 (0.70–0.73)	<0.001***	0.89 (0.85–0.93)	<0.001***	0.96 (0.92–1.01)	0.087
Visuospatial ability	0.25 (0.23–0.27)	<0.001***	0.78 (0.69–0.90)	<0.001***	0.98 (0.85–1.13)	0.774

* *P < 0.05;*

** *P < 0.01;*

*** *P < 0.001. Unadjusted model: without adjustment for any covariates. Model 1 was adjusted for covariates including age and gender. Model 2 was adjusted for covariates including age, gender, marital status, smoking, physical disabilities, intellectual disability, night sleep duration, depression, chronic diseases, and history of falls*.

### Subgroup Analysis of Cognition and Subsequent Falls

In subgroup analysis, we investigated the associations between cognition and falls in the subgroups divided based on different demographic characteristics (including age, gender, location, and education level) after adjusting for all of the covariates listed above. The associations between cognition and falls in the subgroups of people aged 60–74 years, male, or with a lower education level were statistically significant, while significance was not found in the subgroups of people aged ≥75 years, female, or with a higher education level. As shown in Table 6, in the subgroup of people aged 60–74 years, the adjusted OR value was 0.97 (95% CI 0.95–0.99, *P* = 0.008). In the male subgroup, the adjusted OR value was 0.96 (95% CI 0.93–0.98, *P* = 0.001). In the subgroup of people with a lower education level (primary school or below), the adjusted OR value was 0.97 (95% CI 0.95–0.99, *P* = 0.001). As for the subgroups divided according to location, the OR values showed statistically significant associations in both urban (adjusted OR = 0.97, 95% CI 0.95–0.99, *P* = 0.001) and rural residents (adjusted OR = 0.97, 95% CI 0.95–0.99, *P* = 0.003).

**Table 6:** Logistic regression analysis of cognitive function and subsequent falls among subgroups.

	**OR (95% CI)**	***P*-value**
Full sample (*n* = 5,110)	0.97 (0.95–0.99)	0.001**
**Age**		
60–74 (*n* = 4,450)	0.97 (0.95–0.99)	0.008**
≥75 (*n* = 660)	1.00 (0.95–1.05)	0.956
**Gender**		
Male (*n* = 2,794)	0.96 (0.93–0.98)	0.001**
Female (*n* = 2,316)	0.98 (0.96–1.01)	0.134
**Location**		
Urban areas (*n* = 761)	0.97 (0.95–0.99)	0.001**
Rural areas (*n* = 3,672)	0.97 (0.95–0.99)	0.003**
Other (*n* = 677)	0.96 (0.91–1.01)	0.136
**Education level**		
Primary school or below (*n* = 3,841)	0.97 (0.95–0.99)	0.001**
Middle school (*n* = 842)	0.97 (0.91–1.03)	0.265
High school or above (*n* = 427)	1.00 (0.91–1.09)	0.915

** *P < 0.01. Adjusted covariates: age, gender, marital status, smoking, physical disabilities, intellectual disability, night sleep duration, depression, chronic diseases, and history of falls*.

## Discussion

To the best of our knowledge, this was the first study to investigate the longitudinal association between cognitive impairment and subsequent falls in older adults by conducting a subgroup analysis based on a large Chinese population. Subgroup analysis was also performed to compare the associations between the main cognitive domains and falls. The major findings of this study were as follows: first, we confirmed the significant association between cognition and subsequent falls among the Chinese elderly individuals; second, orientation and memory were two cognitive domains that were significantly associated with falls after adjusting for various confounding factors; third, the associations between cognition and falls varied depending on different characteristics of older adults.

The incidence of falls is high among the Chinese elderly. According to a systematic review, the annual fall rate in Chinese older adults ranged from 14.7 to 34%, with a median of 18% (24). In another prospective longitudinal study, the incidence and recurrence of falls in Chinese older adults were 19.3 and 4.75%, respectively (25). In the current study, the incidence of falls was 21.39%, which was consistent with the literature. The high prevalence of falls in older adults highlights the significance of our study.

Previous studies have documented that there was a strong association between cognitive impairment and falls. The prevalence of falls is twice as high among older adults with cognitive impairment than in controls, along with an increased risk of fall-related injury as well as a worse prognosis (12, 26, 27). By analyzing multiple datasets, Susan W Muir et al. (28) showed that cognitive impairment was a risk factor for falls. Yajun Ma et al. (29) pointed out a significant correlation between cognitive impairment and repeated falls. Moreover, Donald S Lipardo et al. (30) reported that older adults with mild cognitive impairment (MCI) were at an increased risk of falls. Importantly, cognitive function training can effectively reduce the incidence of falls. In the current study, we found a strong association between cognitive impairment and subsequent falls after adjusting for multiple risk factors, which is consistent with most of the previous studies.

Dementia is the most severe manifestation of cognitive impairment associated with a higher risk of falls (31). According to the WHO estimates, 5–8% of people aged ≥60 years suffer from dementia; this proportion rises to nearly 30% in people aged ≥85 years (32). In contrast to dementia with psychiatric behavioral abnormalities and significant decline in the capacity to perform activities of daily living, MCI patients only exhibit mild cognitive decline, and their independent conduction of daily activities is not significantly affected. Previous studies have found that cognition can return to normal levels in 20–30% of MCI patients, while about 50% of individuals with MCI develop dementia within 5 years (33). Therefore, MCI is considered the optimal intervention window for dementia, and a predictor and potentially modifiable risk factor of falls (34). Early identification and intervention at the stage of MCI may be an important way to reduce the incidence of falls in the elderly. The evaluation of cognitive function in this study was based on the cognitive scale of the CHARLS questionnaire. The cognitive score was a continuous variable with a maximum total score of 21 points. The results showed that each one-point increase in cognitive function would have a significant positive impact on the outcome of falls, suggesting that early intervention for cognitive decline has practical significance in preventing falls in the elderly.

Given that the preclinical stage of Alzheimer's disease can be predicted by evaluating the main cognitive domains (15), many studies have explored the associations between cognitive domains and falls. Veronika van der Wardt et al. (18) demonstrated through hierarchical regression analysis that the incidence of falls was significantly associated with spatial memory abilities. Inconsistently, Roee Holtzer et al. (17) found that worse performance in speed/executive attention showed a significant association with a higher risk of both single and repeated falls, while the memory was associated with neither single nor repeated falls. Tuo Yu Chen et al. (13) also found that processing speed and executive function were significantly associated with falls and repeated falls. Other studies have found that impaired executive function may reduce an individual's ability to focus on the sensory information related to maintaining balance while walking, thereby increasing the risk of falls (34). Emma O'Keeffe et al. (35) pointed out that the severity of temporal orientation disorder was closely related to the severity of dementia, and cognitive impairment diagnosis by temporal orientation assessment yielded high sensitivity and specificity. Our results showed that both orientation and memory exhibited strong associations with fall outcomes. Accordingly, orientation tests may have huge prospects for clinical application as a simple and effective screening method for assessing fall risks in older adults. However, further studies are needed to investigate the value of these cognitive domains in predicting falls.

Our study also demonstrated that the associations between cognition and falls varied among different subgroups defined by multiple demographic characteristics of older adults. Thus, appropriate predictors should be considered when assessing the risk of falls among the elderly with different basic characteristics and health statuses. Jae-Hyun Kim (36) pointed out that screening for cognitive impairment in people with a history of falls may help prevent the occurrence of falls, especially in men aged ≤64 years, which suggested that the association between cognition and falls was age-dependent. In this study, we found that the association between cognitive function and subsequent falls was significant in individuals aged 60–74 years. The findings from a study by Deborah A Jehu et al. (37) suggested that improving cognitive function in older men can reduce the risk of falls. Consistently, cognitive function scores of older men showed a strong association with subsequent falls in our study. Among older adults who lived in different areas, our model showed a wide range of applicability, with statistical significance in both urban and rural residents. An interesting finding was that the improvement of cognitive function was more significant in the prevention of falls among older adults with an education level of primary school or below. It may be due to the greater variability in cognitive function in the subgroup of people with lower education levels compared with other groups. The results obtained in the subgroup analysis may provide a basis for age-specific, sex-specific, and education-specific fall prevention strategies in older adults. Our findings could supplement the available evidence on fall risk assessment by providing more accurate, evidence-based prevention and intervention measures for specific high-risk populations. Nevertheless, the related research is still scarce and further studies are needed to better understand the associations between cognition and falls in selected high-risk populations.

There were also some limitations in this study. First, only the Chinese elderly population was investigated in this study, which may limit the generalizability of the study. Second, the evaluation of cognitive impairment in this study was based on the questionnaire indicators in CHARLS, which may have led to evaluation bias in cognitive impairment since no other cognitive function evaluation scales were adopted. Finally, the collection of the study information was mainly based on the respondents' self-reports, and the validity of self-reported fall results was not tested. Accordingly, the possibility of recall bias cannot be excluded in this study.

## Conclusions

The results of the present study demonstrated a significant association between cognition and subsequent falls among Chinese older adults. Furthermore, assessment of orientation and memory might be an effective approach for early identification of older adults at risk of falls. Finally, attention should be paid to the specificity of subgroups with different demographic characteristics in fall risk assessment to design more accurate prevention and intervention measures for specific high-risk populations.

## Data Availability Statement

The datasets for this study can be found in the China Health and Retirement Longitudinal Study (CHARLS). The current study is a secondary analysis of public data of CHARLS. The original datasets of CHARLS is accessible on http://charls.pku.edu.cn/en.

## Ethics Statement

The studies involving human participants were reviewed and approved by Biomedical Ethics Review Committee of Peking University. The patients/participants provided their written informed consent to participate in this study. Written informed consent was obtained from the individual(s) for the publication of any potentially identifiable images or data included in this article.

## Author Contributions

RZ contributed to conceptualization and writing the original draft preparation. JL conducted data analysis. RZ, JL, and MC revised the manuscript. All authors contributed to the article and approved the submitted version.

## Funding

This research was supported by Zhejiang Provincial Natural Science Foundation of China under Grant No. LGF19H260006. All sponsors had no role in the study design, data collection, and analysis, decision to publish, or preparation of the manuscript.

## Conflict of Interest

The authors declare that the research was conducted in the absence of any commercial or financial relationships that could be construed as a potential conflict of interest.

## Publisher's Note

All claims expressed in this article are solely those of the authors and do not necessarily represent those of their affiliated organizations, or those of the publisher, the editors and the reviewers. Any product that may be evaluated in this article, or claim that may be made by its manufacturer, is not guaranteed or endorsed by the publisher.
