# Supplementary material for: The Association Between Cognitive Impairment and Subsequent Falls Among Older Adults: Evidence From the China Health and Retirement Longitudinal Study
Source: Front Public Health. 2022 Jun 15;10:900315. doi: 10.3389/fpubh.2022.900315 (PMC9240660; doi:10.3389/fpubh.2022.900315)
Supplement: Supplementary file 1 [file Data_Sheet_1.docx]

Supplementary Material

**Supplementary Table 1 |** Logistic regression analysis in the full sample.

| **Model** | **Variables** | ***P*** | **95%CI** |
| --- | --- | --- | --- |
| Model 1 | Gender (female) | <0.001 | 1.43 (1.20-1.69) |
|  | Age | 0.003 | 1.01 (1.00-1.01) |
|  | Marital status (other) | 0.064 | 1.18 (0.99-1.40) |
|  | Smoking (no) | 0.537 | 0.95 (0.79-1.13) |
|  | Physical disabilities (no) | <0.001 | 0.55 (0.44-0.69) |
|  | Intellectual disability (no) | 0.004 | 0.70 (0.55-0.89) |
|  | Night sleep duration | 0.002 |  |
|  | Night sleep duration (6~8 h) | 0.001 | 0.76 (0.66-0.89) |
|  | Night sleep duration (>8 h) | 0.306 | 0.88 (0.68-1.13) |
|  | CES-D (≥10) | 0.002 | 1.26 (1.09-1.45) |
|  | Chronic diseases (≥1) | 0.35 | 1.09 (0.91-1.30) |
|  | History of falls (no) | <0.001 | 0.34 (0.29-0.40) |
|  | Cognition | 0.001 | 0.97 (0.95-0.99) |
| Model 2 | Gender (female) | <0.001 | 1.44 (1.21-1.71) |
|  | Age | 0.008 | 1.01 (1.00-1.01) |
|  | Marital status (other) | 0.042 | 1.20 (1.01-1.42) |
|  | Smoking (no) | 0.525 | 0.94 (0.79-1.13) |
|  | Physical disabilities (no) | <0.001 | 0.54 (0.43-0.68) |
|  | Intellectual disability (no) | 0.002 | 0.68 (0.54-0.87) |
|  | Night sleep duration | 0.001 |  |
|  | Night sleep duration (6~8 h) | <0.001 | 0.76 (0.65-0.88) |
|  | Night sleep duration (>8 h) | 0.347 | 0.89 (0.69-1.14) |
|  | CES-D (≥10) | 0.001 | 1.27 (1.09-1.46) |
|  | Chronic diseases (≥1) | 0.443 | 1.07 (0.90-1.28) |
|  | History of falls (no) | <0.001 | 0.34 (0.29-0.39) |
|  | Orientation | 0.013 | 0.94 (0.90-0.99) |
| Model 3 | Gender (female) | <0.001 | 1.48 (1.25-1.76) |
|  | Age | 0.008 | 1.01 (1.00-1.01) |
|  | Marital status (other) | 0.067 | 1.18 (0.99-1.40) |
|  | Smoking (no) | 0.534 | 0.95 (0.79-1.13) |
|  | Physical disabilities (no) | <0.001 | 0.55 (0.44-0.69) |
|  | Intellectual disability (no) | 0.003 | 0.70 (0.55-0.89) |
|  | Night sleep duration | 0.001 |  |
|  | Night sleep duration (6~8 h) | <0.001 | 0.76 (0.65-0.88) |
|  | Night sleep duration (>8 h) | 0.316 | 0.88 (0.68-1.13) |
|  | CES-D (≥10) | 0.002 | 1.26 (1.09-1.46) |
|  | Chronic diseases (≥1) | 0.371 | 1.08 (0.91-1.29) |
|  | History of falls (no) | <0.001 | 0.34 (0.29-0.39) |
|  | Memory | 0.001 | 0.93 (0.90-0.97) |
| Model 4 | Gender (female) | <0.001 | 1.44 (1.21-1.71) |
|  | Age | 0.019 | 1.01 (1.00-1.01) |
|  | Marital status (other) | 0.038 | 1.20 (1.01-1.43) |
|  | Smoking (no) | 0.473 | 0.94 (0.78-1.12) |
|  | Physical disabilities (no) | <0.001 | 0.54 (0.43-0.68) |
|  | Intellectual disability (no) | 0.002 | 0.68 (0.53-0.86) |
|  | Night sleep duration | 0.001 |  |
|  | Night sleep duration (6~8 h) | <0.001 | 0.75 (0.65-0.88) |
|  | Night sleep duration (>8 h) | 0.382 | 0.89 (0.69-1.15) |
|  | CES-D (≥10) | 0.001 | 1.27 (1.10-1.47) |
|  | Chronic diseases (≥1) | 0.442 | 1.07 (0.90-1.28) |
|  | History of falls (no) | <0.001 | 0.34 (0.29-0.39) |
|  | Calculation | 0.087 | 0.96 (0.92-1.01) |
| Model 5 | Gender (female) | <0.001 | 1.47 (1.24-1.75) |
|  | Age | 0.051 | 1.01 (1.00-1.01) |
|  | Marital status (other) | 0.031 | 1.21 (1.02-1.44) |
|  | Smoking (no) | 0.453 | 0.93 (0.78-1.12) |
|  | Physical disabilities (no) | <0.001 | 0.53 (0.43-0.67) |
|  | Intellectual disability (no) | 0.001 | 0.67 (0.52-0.85) |
|  | Night sleep duration | 0.001 |  |
|  | Night sleep duration (6~8 h) | <0.001 | 0.74 (0.64-0.87) |
|  | Night sleep duration (>8 h) | 0.411 | 0.90 (0.70-1.16) |
|  | CES-D (≥10) | 0.001 | 1.28 (1.11-1.48) |
|  | Chronic diseases (≥1) | 0.516 | 1.06 (0.89-1.26) |
|  | History of falls (no) | <0.001 | 0.33 (0.28-0.39) |
|  | Visuospatial ability (pass) | 0.774 | 0.98 (0.85-1.13) |

**Supplementary Table 2 |** Logistic regression analysis in subgroups divided according to gender.

| **Model** | **Variables** | **Female** | | **Male** | |
| --- | --- | --- | --- | --- | --- |
|  |  | ***P*** | **95%CI** | ***P*** | **95%CI** |
| Model 1 | Age | 0.109 | 1.01 (1.00-1.02) | 0.001 | 1.01 (1.01-1.02) |
|  | Marital status (other) | 0.153 | 1.18 (0.94-1.48) | 0.279 | 1.16 (0.89-1.53) |
|  | Smoking (no) | 0.939 | 0.99 (0.70-1.40) | 0.596 | 0.95 (0.77-1.16) |
|  | Physical disabilities (no) | 0.007 | 0.62 (0.44-0.88) | <0.001 | 0.51 (0.37-0.68) |
|  | Intellectual disability (no) | 0.128 | 0.75 (0.52-1.09) | 0.022 | 0.68 (0.49-0.95) |
|  | Night sleep duration | 0.003 |  | 0.03 |  |
|  | Night sleep duration (6~8 h) | 0.004 | 0.74 (0.60-0.91) | 0.103 | 0.83 (0.66-1.04) |
|  | Night sleep duration (>8 h) | 0.013 | 0.61 (0.41-0.90) | 0.232 | 1.23 (0.87-1.74) |
|  | CES-D (≥10) | 0.003 | 1.36 (1.11-1.66) | 0.184 | 1.16 (0.93-1.43) |
|  | Chronic diseases (≥1) | 0.07 | 1.28 (0.98-1.67) | 0.708 | 0.96 (0.75-1.21) |
|  | History of falls (no) | <0.001 | 0.32 (0.26-0.40) | <0.001 | 0.37 (0.29-0.47) |
|  | Cognition | 0.134 | 0.98 (0.96-1.01) | 0.001 | 0.96 (0.93-0.98) |
| Model 2 | Age | 0.148 | 1.01 (1.00-1.02) | 0.002 | 1.01 (1.00-1.02) |
|  | Marital status (other) | 0.125 | 1.20 (0.95-1.50) | 0.216 | 1.19 (0.91-1.56) |
|  | Smoking (no) | 0.878 | 0.97 (0.69-1.38) | 0.598 | 0.95 (0.77-1.16) |
|  | Physical disabilities (no) | 0.005 | 0.61 (0.43-0.87) | <0.001 | 0.50 (0.37-0.67) |
|  | Intellectual disability (no) | 0.106 | 0.74 (0.52-1.07) | 0.013 | 0.66 (0.48-0.92) |
|  | Night sleep duration | 0.003 |  | 0.021 |  |
|  | Night sleep duration (6~8 h) | 0.003 | 0.73 (0.59-0.90) | 0.087 | 0.82 (0.65-1.03) |
|  | Night sleep duration (>8 h) | 0.016 | 0.62 (0.42-0.91) | 0.218 | 1.24 (0.88-1.76) |
|  | CES-D (≥10) | 0.002 | 1.37 (1.12-1.67) | 0.164 | 1.16 (0.94-1.44) |
|  | Chronic diseases (≥1) | 0.085 | 1.26 (0.97-1.64) | 0.599 | 0.94 (0.74-1.19) |
|  | History of falls (no) | <0.001 | 0.32 (0.26-0.39) | <0.001 | 0.36 (0.29-0.46) |
|  | Orientation | 0.448 | 0.98 (0.92-1.04) | 0.003 | 0.89 (0.83-0.96) |
| Model 3 | Age | 0.118 | 1.01 (1.00-1.02) | 0.005 | 1.01 (1.00-1.02) |
|  | Marital status (other) | 0.165 | 1.18 (0.94-1.48) | 0.276 | 1.16 (0.89-1.53) |
|  | Smoking (no) | 0.936 | 0.99 (0.70-1.40) | 0.599 | 0.95 (0.77-1.16) |
|  | Physical disabilities (no) | 0.007 | 0.62 (0.44-0.88) | <0.001 | 0.50 (0.37-0.67) |
|  | Intellectual disability (no) | 0.13 | 0.76 (0.53-1.09) | 0.014 | 0.66 (0.48-0.92) |
|  | Night sleep duration | 0.003 |  | 0.019 |  |
|  | Night sleep duration (6~8 h) | 0.003 | 0.74 (0.60-0.90) | 0.07 | 0.81 (0.65-1.02) |
|  | Night sleep duration (>8 h) | 0.013 | 0.61 (0.42-0.90) | 0.246 | 1.23 (0.87-1.73) |
|  | CES-D (≥10) | 0.002 | 1.36 (1.12-1.66) | 0.174 | 1.16 (0.94-1.44) |
|  | Chronic diseases (≥1) | 0.068 | 1.28 (0.98-1.67) | 0.635 | 0.94 (0.75-1.20) |
|  | History of falls (no) | <0.001 | 0.32 (0.26-0.40) | <0.001 | 0.36 (0.29-0.46) |
|  | Memory | 0.084 | 0.95 (0.90-1.01) | 0.002 | 0.92 (0.87-0.97) |
| Model 4 | Age | 0.145 | 1.01 (1.00-1.02) | 0.013 | 1.01 (1.00-1.02) |
|  | Marital status (other) | 0.127 | 1.19 (0.95-1.50) | 0.213 | 1.19 (0.91-1.56) |
|  | Smoking (no) | 0.892 | 0.98 (0.69-1.38) | 0.505 | 0.93 (0.76-1.15) |
|  | Physical disabilities (no) | 0.006 | 0.62 (0.44-0.87) | <0.001 | 0.49 (0.36-0.66) |
|  | Intellectual disability (no) | 0.11 | 0.74 (0.52-1.07) | 0.007 | 0.64 (0.46-0.88) |
|  | Night sleep duration | 0.003 |  | 0.012 |  |
|  | Night sleep duration (6~8 h) | 0.003 | 0.73 (0.60-0.90) | 0.064 | 0.81 (0.64-1.01) |
|  | Night sleep duration (>8 h) | 0.015 | 0.62 (0.42-0.91) | 0.192 | 1.26 (0.89-1.78) |
|  | CES-D (≥10) | 0.002 | 1.37 (1.12-1.67) | 0.15 | 1.17 (0.95-1.45) |
|  | Chronic diseases (≥1) | 0.077 | 1.27 (0.98-1.66) | 0.558 | 0.93 (0.74-1.18) |
|  | History of falls (no) | <0.001 | 0.32 (0.26-0.40) | <0.001 | 0.36 (0.28-0.45) |
|  | Calculation | 0.259 | 0.97 (0.91-1.03) | 0.178 | 0.95 (0.89-1.02) |
| Model 5 | Age | 0.192 | 1.01 (1.00-1.02) | 0.031 | 1.01 (1.00-1.02) |
|  | Marital status (other) | 0.113 | 1.20 (0.96-1.51) | 0.198 | 1.20 (0.91-1.57) |
|  | Smoking (no) | 0.831 | 0.96 (0.68-1.36) | 0.502 | 0.93 (0.76-1.15) |
|  | Physical disabilities (no) | 0.004 | 0.61 (0.43-0.86) | <0.001 | 0.48 (0.36-0.65) |
|  | Intellectual disability (no) | 0.095 | 0.73 (0.51-1.06) | 0.004 | 0.62 (0.45-0.86) |
|  | Night sleep duration | 0.003 |  | 0.009 |  |
|  | Night sleep duration (6~8 h) | 0.002 | 0.72 (0.59-0.89) | 0.05 | 0.80 (0.64-1.00) |
|  | Night sleep duration (>8 h) | 0.019 | 0.63 (0.43-0.93) | 0.197 | 1.26 (0.89-1.77) |
|  | CES-D (≥10) | 0.002 | 1.38 (1.13-1.68) | 0.134 | 1.18 (0.95-1.46) |
|  | Chronic diseases (≥1) | 0.091 | 1.26 (0.96-1.64) | 0.494 | 0.92 (0.73-1.17) |
|  | History of falls (no) | <0.001 | 0.32 (0.26-0.39) | <0.001 | 0.36 (0.28-0.45) |
|  | Visuospatial ability (pass) | 0.973 | 1.00 (0.83-1.22) | 0.774 | 0.97 (0.78-1.20) |

**Supplementary Table 3 |** Logistic regression analysis in subgroups divided according to age.

| **Model** | **Variables** | **Aged >75** | | **Aged 60-74** | |
| --- | --- | --- | --- | --- | --- |
|  |  | ***P*** | **95%CI** | ***P*** | **95%CI** |
| Model 1 | Gender (female) | 0.778 | 1.06 (0.70-1.61) | <0.001 | 1.55 (1.28-1.88) |
|  | Marital status (other) | 0.327 | 1.21 (0.83-1.78) | 0.166 | 1.15 (0.94-1.40) |
|  | Smoking (no) | 0.666 | 0.91 (0.61-1.38) | 0.558 | 0.94 (0.77-1.15) |
|  | Physical disabilities (no) | 0.423 | 0.78 (0.42-1.44) | <0.001 | 0.54 (0.42-0.69) |
|  | Intellectual disability (no) | 0.526 | 0.82 (0.43-1.53) | 0.022 | 0.73 (0.56-0.96) |
|  | Night sleep duration | 0.107 |  | 0.037 |  |
|  | Night sleep duration (6~8 h) | 0.035 | 0.66 (0.45-0.97) | 0.011 | 0.81 (0.68-0.95) |
|  | Night sleep duration (>8 h) | 0.419 | 0.79 (0.45-1.39) | 0.592 | 0.92 (0.69-1.23) |
|  | CES-D (≥10) | 0.946 | 1.01 (0.70-1.48) | <0.001 | 1.34 (1.14-1.57) |
|  | Chronic diseases (≥1) | 0.375 | 0.83 (0.55-1.25) | 0.078 | 1.20 (0.98-1.46) |
|  | History of falls (no) | <0.001 | 0.47 (0.32-0.71) | <0.001 | 0.32 (0.27-0.39) |
|  | Cognition | 0.956 | 1.00 (0.95-1.05) | 0.008 | 0.97 (0.95-0.99) |
|  | Age | 0.465 | 1.01 (0.99-1.02) | 0.149 | 1.01 (1.00-1.01) |
| Model 2 | Gender (female) | 0.729 | 1.08 (0.71-1.63) | <0.001 | 1.56 (1.29-1.90) |
|  | Marital status (other) | 0.308 | 1.22 (0.83-1.79) | 0.145 | 1.16 (0.95-1.41) |
|  | Smoking (no) | 0.652 | 0.91 (0.60-1.37) | 0.555 | 0.94 (0.77-1.15) |
|  | Physical disabilities (no) | 0.396 | 0.77 (0.41-1.42) | <0.001 | 0.54 (0.42-0.69) |
|  | Intellectual disability (no) | 0.528 | 0.82 (0.44-1.53) | 0.016 | 0.72 (0.55-0.94) |
|  | Night sleep duration | 0.1 |  | 0.029 |  |
|  | Night sleep duration (6~8 h) | 0.032 | 0.66 (0.45-0.97) | 0.009 | 0.80 (0.68-0.95) |
|  | Night sleep duration (>8 h) | 0.432 | 0.80 (0.46-1.40) | 0.627 | 0.93 (0.70-1.24) |
|  | CES-D (≥10) | 0.905 | 1.02 (0.71-1.48) | <0.001 | 1.35 (1.15-1.58) |
|  | Chronic diseases (≥1) | 0.363 | 0.83 (0.55-1.25) | 0.094 | 1.19 (0.97-1.45) |
|  | History of falls (no) | <0.001 | 0.47 (0.32-0.71) | <0.001 | 0.32 (0.27-0.38) |
|  | Age | 0.538 | 1.00 (0.99-1.02) | 0.234 | 1.00 (1.00-1.01) |
|  | Orientation | 0.742 | 1.02 (0.91-1.15) | 0.024 | 0.94 (0.89-0.99) |
| Model 3 | Gender (female) | 0.771 | 1.06 (0.70-1.61) | <0.001 | 1.61 (1.33-1.95) |
|  | Marital status (other) | 0.356 | 1.20 (0.82-1.76) | 0.176 | 1.15 (0.94-1.40) |
|  | Smoking (no) | 0.674 | 0.92 (0.61-1.38) | 0.558 | 0.94 (0.77-1.15) |
|  | Physical disabilities (no) | 0.445 | 0.79 (0.42-1.46) | <0.001 | 0.54 (0.42-0.69) |
|  | Intellectual disability (no) | 0.513 | 0.81 (0.43-1.52) | 0.019 | 0.73 (0.55-0.95) |
|  | Night sleep duration | 0.111 |  | 0.027 |  |
|  | Night sleep duration (6~8 h) | 0.036 | 0.67 (0.46-0.98) | 0.008 | 0.80 (0.68-0.94) |
|  | Night sleep duration (>8 h) | 0.382 | 0.78 (0.44-1.37) | 0.619 | 0.93 (0.70-1.24) |
|  | CES-D (≥10) | 0.987 | 1.00 (0.69-1.46) | <0.001 | 1.34 (1.14-1.57) |
|  | Chronic diseases (≥1) | 0.384 | 0.83 (0.55-1.26) | 0.082 | 1.20 (0.98-1.46) |
|  | History of falls (no) | <0.001 | 0.47 (0.32-0.71) | <0.001 | 0.32 (0.27-0.38) |
|  | Age | 0.365 | 1.01 (0.99-1.02) | 0.267 | 1.00 (1.00-1.01) |
|  | Memory | 0.571 | 0.97 (0.88-1.07) | 0.007 | 0.94 (0.90-0.98) |
| Model 4 | Gender (female) | 0.72 | 1.08 (0.71-1.65) | <0.001 | 1.57 (1.29-1.91) |
|  | Marital status (other) | 0.307 | 1.22 (0.83-1.79) | 0.138 | 1.16 (0.95-1.42) |
|  | Smoking (no) | 0.662 | 0.91 (0.61-1.37) | 0.51 | 0.94 (0.77-1.14) |
|  | Physical disabilities (no) | 0.401 | 0.77 (0.41-1.42) | <0.001 | 0.53 (0.42-0.68) |
|  | Intellectual disability (no) | 0.534 | 0.82 (0.44-1.54) | 0.013 | 0.71 (0.54-0.93) |
|  | Night sleep duration | 0.102 |  | 0.026 |  |
|  | Night sleep duration (6~8 h) | 0.033 | 0.66 (0.45-0.97) | 0.008 | 0.80 (0.68-0.94) |
|  | Night sleep duration (>8 h) | 0.433 | 0.80 (0.46-1.40) | 0.677 | 0.94 (0.71-1.25) |
|  | CES-D (≥10) | 0.893 | 1.03 (0.70-1.50) | <0.001 | 1.36 (1.16-1.59) |
|  | Chronic diseases (≥1) | 0.371 | 0.83 (0.55-1.25) | 0.088 | 1.19 (0.97-1.45) |
|  | History of falls (no) | <0.001 | 0.47 (0.32-0.71) | <0.001 | 0.32 (0.27-0.38) |
|  | Age | 0.541 | 1.00 (0.99-1.02) | 0.422 | 1.00 (1.00-1.01) |
|  | Calculation | 0.762 | 1.02 (0.91-1.13) | 0.163 | 0.97 (0.92-1.02) |
| Model 5 | Gender (female) | 0.826 | 1.05 (0.69-1.59) | <0.001 | 1.62 (1.33-1.96) |
|  | Marital status (other) | 0.33 | 1.21 (0.83-1.77) | 0.129 | 1.17 (0.96-1.42) |
|  | Smoking (no) | 0.683 | 0.92 (0.61-1.38) | 0.501 | 0.93 (0.76-1.14) |
|  | Physical disabilities (no) | 0.43 | 0.78 (0.42-1.45) | <0.001 | 0.53 (0.41-0.68) |
|  | Intellectual disability (no) | 0.54 | 0.82 (0.44-1.54) | 0.009 | 0.70 (0.54-0.91) |
|  | Night sleep duration | 0.11 |  | 0.016 |  |
|  | Night sleep duration (6~8 h) | 0.036 | 0.67 (0.46-0.97) | 0.005 | 0.79 (0.67-0.93) |
|  | Night sleep duration (>8 h) | 0.398 | 0.78 (0.45-1.38) | 0.733 | 0.95 (0.71-1.27) |
|  | CES-D (≥10) | 0.957 | 1.01 (0.70-1.46) | <0.001 | 1.37 (1.17-1.60) |
|  | Chronic diseases (≥1) | 0.374 | 0.83 (0.55-1.25) | 0.105 | 1.18 (0.97-1.44) |
|  | History of falls (no) | <0.001 | 0.47 (0.32-0.71) | <0.001 | 0.32 (0.27-0.38) |
|  | Age | 0.409 | 1.01 (0.99-1.02) | 0.797 | 1.00 (1.00-1.01) |
|  | Visuospatial ability (pass) | 0.69 | 0.93 (0.65-1.33) | 0.668 | 1.04 (0.88-1.22) |

**Supplementary Table 4 |** Logistic regression analysis in subgroups divided according to location.

| **Model** | **Variables** | **Urban areas** | | **Rural areas** | | **Other** | |
| --- | --- | --- | --- | --- | --- | --- | --- |
|  |  | ***P*** | **95%CI** | ***P*** | **95%CI** | ***P*** | **95%CI** |
| Model 1 | Gender (female) | 0.006 | 1.33 (1.09-1.62) | <0.001 | 1.61 (1.31-1.98) | 0.179 | 0.72 (0.44-1.16) |
|  | Age | 0.003 | 1.01 (1.00-1.02) | 0.019 | 1.01 (1.00-1.01) | 0.136 | 1.01 (1.00-1.03) |
|  | Marital status (other) | 0.198 | 1.14 (0.94-1.38) | 0.899 | 0.99 (0.8-1.22) | 0.039 | 1.64 (1.03-2.61) |
|  | Smoking (no) | 0.834 | 1.02 (0.83-1.26) | 0.44 | 0.92 (0.75-1.14) | 0.57 | 1.15 (0.70-1.90) |
|  | Physical disabilities (no) | <0.001 | 0.55 (0.43-0.71) | <0.001 | 0.53 (0.41-0.69) | 0.808 | 0.92 (0.47-1.80) |
|  | Intellectual disability (no) | 0.009 | 0.69 (0.53-0.91) | 0.037 | 0.74 (0.56-0.98) | 0.216 | 0.64 (0.32-1.30) |
|  | Night sleep duration | 0.007 |  | <0.001 |  | 0.234 |  |
|  | Night sleep duration (6~8 h) | 0.002 | 0.76 (0.64-0.90) | <0.001 | 0.69 (0.57-0.82) | 0.546 | 0.88 (0.57-1.35) |
|  | Night sleep duration (>8 h) | 0.268 | 0.85 (0.64-1.13) | 0.596 | 0.93 (0.70-1.23) | 0.09 | 0.43 (0.17-1.14) |
|  | CES-D (≥10) | <0.001 | 1.41 (1.20-1.66) | <0.001 | 1.38 (1.16-1.64) | 0.785 | 1.06 (0.70-1.61) |
|  | Chronic diseases (≥1) | 0.972 | 1.00 (0.82-1.23) | 0.656 | 1.05 (0.85-1.29) | 0.799 | 0.94 (0.58-1.52) |
|  | History of falls (no) | <0.001 | 0.33 (0.28-0.40) | <0.001 | 0.36 (0.30-0.43) | <0.001 | 0.25 (0.16-0.40) |
|  | Cognition | 0.001 | 0.97 (0.95-0.99) | 0.003 | 0.97 (0.95-0.99) | 0.136 | 0.96 (0.91-1.01) |
| Model 2 | Gender (female) | 0.004 | 1.34 (1.10-1.64) | <0.001 | 1.63 (1.33-2.00) | 0.196 | 0.73 (0.45-1.18) |
|  | Age | 0.009 | 1.01 (1.00-1.01) | 0.04 | 1.01 (1.00-1.01) | 0.319 | 1.01 (0.99-1.02) |
|  | Marital status (other) | 0.133 | 1.16 (0.96-1.41) | 0.959 | 1.01 (0.81-1.24) | 0.035 | 1.66 (1.04-2.64) |
|  | Smoking (no) | 0.853 | 1.02 (0.83-1.26) | 0.439 | 0.92 (0.75-1.14) | 0.62 | 1.13 (0.69-1.86) |
|  | Physical disabilities (no) | <0.001 | 0.54 (0.42-0.69) | <0.001 | 0.53 (0.41-0.68) | 0.796 | 0.92 (0.47-1.79) |
|  | Intellectual disability (no) | 0.004 | 0.67 (0.51-0.88) | 0.024 | 0.72 (0.55-0.96) | 0.146 | 0.60 (0.30-1.20) |
|  | Night sleep duration | 0.004 |  | <0.001 |  | 0.269 |  |
|  | Night sleep duration (6~8 h) | 0.001 | 0.75 (0.63-0.89) | <0.001 | 0.68 (0.57-0.81) | 0.43 | 0.84 (0.55-1.29) |
|  | Night sleep duration (>8 h) | 0.308 | 0.86 (0.65-1.14) | 0.633 | 0.93 (0.71-1.24) | 0.115 | 0.46 (0.18-1.21) |
|  | CES-D (≥10) | <0.001 | 1.42 (1.21-1.67) | <0.001 | 1.39 (1.17-1.65) | 0.704 | 1.08 (0.71-1.65) |
|  | Chronic diseases (≥1) | 0.875 | 0.98 (0.81-1.20) | 0.78 | 1.03 (0.84-1.26) | 0.693 | 0.91 (0.56-1.46) |
|  | History of falls (no) | <0.001 | 0.33 (0.28-0.39) | <0.001 | 0.36 (0.30-0.43) | <0.001 | 0.25 (0.16-0.38) |
|  | Orientation | 0.02 | 0.94 (0.89-0.99) | 0.027 | 0.94 (0.89-0.99) | 0.713 | 0.97 (0.83-1.14) |
| Model 3 | Gender (female) | 0.001 | 1.40 (1.15-1.71) | <0.001 | 1.69 (1.38-2.07) | 0.255 | 0.76 (0.47-1.23) |
|  | Age | 0.006 | 1.01 (1.00-1.01) | 0.033 | 1.01 (1.00-1.01) | 0.175 | 1.01 (1.00-1.03) |
|  | Marital status (other) | 0.222 | 1.13 (0.93-1.37) | 0.882 | 0.98 (0.80-1.22) | 0.043 | 1.62 (1.02-2.59) |
|  | Smoking (no) | 0.847 | 1.02 (0.83-1.26) | 0.441 | 0.92 (0.75-1.14) | 0.594 | 1.15 (0.70-1.88) |
|  | Physical disabilities (no) | <0.001 | 0.54 (0.42-0.70) | <0.001 | 0.53 (0.41-0.69) | 0.762 | 0.90 (0.46-1.77) |
|  | Intellectual disability (no) | 0.008 | 0.69 (0.53-0.91) | 0.032 | 0.74 (0.56-0.97) | 0.265 | 0.67 (0.33-1.36) |
|  | Night sleep duration | 0.005 |  | <0.001 |  | 0.235 |  |
|  | Night sleep duration (6~8 h) | 0.001 | 0.75 (0.63-0.89) | <0.001 | 0.68 (0.57-0.81) | 0.542 | 0.88 (0.57-1.34) |
|  | Night sleep duration (>8 h) | 0.255 | 0.85 (0.64-1.13) | 0.58 | 0.92 (0.7-1.22) | 0.09 | 0.44 (0.17-1.14) |
|  | CES-D (≥10) | <0.001 | 1.42 (1.20-1.67) | <0.001 | 1.38 (1.17-1.64) | 0.76 | 1.07 (0.70-1.62) |
|  | Chronic diseases (≥1) | 0.998 | 1.00 (0.82-1.22) | 0.671 | 1.05 (0.85-1.29) | 0.843 | 0.95 (0.59-1.54) |
|  | History of falls (no) | <0.001 | 0.33 (0.28-0.39) | <0.001 | 0.36 (0.3-0.43) | <0.001 | 0.25 (0.16-0.40) |
|  | Memory | <0.001 | 0.91 (0.87-0.96) | 0.001 | 0.92 (0.88-0.97) | 0.045 | 0.89 (0.79-1.00) |
| Model 4 | Gender (female) | 0.004 | 1.35 (1.10-1.65) | <0.001 | 1.64 (1.33-2.02) | 0.178 | 0.72 (0.44-1.16) |
|  | Age | 0.02 | 1.01 (1.00-1.01) | 0.081 | 1.01 (1.00-1.01) | 0.242 | 1.01 (0.99-1.03) |
|  | Marital status (other) | 0.119 | 1.17 (0.96-1.42) | 0.918 | 1.01 (0.82-1.25) | 0.032 | 1.67 (1.05-2.66) |
|  | Smoking (no) | 0.92 | 1.01 (0.82-1.25) | 0.407 | 0.92 (0.74-1.13) | 0.623 | 1.13 (0.69-1.85) |
|  | Physical disabilities (no) | <0.001 | 0.53 (0.42-0.69) | <0.001 | 0.52 (0.40-0.67) | 0.799 | 0.92 (0.47-1.79) |
|  | Intellectual disability (no) | 0.003 | 0.67 (0.51-0.88) | 0.018 | 0.71 (0.54-0.94) | 0.159 | 0.61 (0.30-1.22) |
|  | Night sleep duration | 0.004 |  | <0.001 |  | 0.249 |  |
|  | Night sleep duration (6~8 h) | 0.001 | 0.75 (0.63-0.89) | <0.001 | 0.68 (0.57-0.81) | 0.456 | 0.85 (0.55-1.30) |
|  | Night sleep duration (>8 h) | 0.335 | 0.87 (0.66-1.15) | 0.688 | 0.95 (0.72-1.25) | 0.102 | 0.45 (0.17-1.17) |
|  | CES-D (≥10) | <0.001 | 1.43 (1.21-1.68) | <0.001 | 1.39 (1.18-1.65) | 0.736 | 1.08 (0.71-1.63) |
|  | Chronic diseases (≥1) | 0.87 | 0.98 (0.81-1.20) | 0.781 | 1.03 (0.84-1.27) | 0.695 | 0.91 (0.57-1.46) |
|  | History of falls (no) | <0.001 | 0.33 (0.28-0.39) | <0.001 | 0.36 (0.30-0.43) | <0.001 | 0.25 (0.16-0.39) |
|  | Calculation | 0.103 | 0.96 (0.91-1.01) | 0.183 | 0.97 (0.92-1.02) | 0.418 | 0.95 (0.83-1.08) |
| Model 5 | Gender (female) | 0.002 | 1.37 (1.12-1.67) | <0.001 | 1.69 (1.37-2.07) | 0.201 | 0.73 (0.45-1.18) |
|  | Age | 0.04 | 1.01 (1.00-1.01) | 0.155 | 1.00 (1.00-1.01) | 0.335 | 1.01 (0.99-1.02) |
|  | Marital status (other) | 0.114 | 1.17 (0.96-1.42) | 0.842 | 1.02 (0.83-1.26) | 0.034 | 1.66 (1.04-2.64) |
|  | Smoking (no) | 0.951 | 1.01 (0.82-1.24) | 0.399 | 0.91 (0.74-1.13) | 0.641 | 1.13 (0.69-1.84) |
|  | Physical disabilities (no) | <0.001 | 0.53 (0.41-0.68) | <0.001 | 0.51 (0.40-0.67) | 0.792 | 0.91 (0.47-1.79) |
|  | Intellectual disability (no) | 0.003 | 0.66 (0.50-0.86) | 0.012 | 0.70 (0.53-0.93) | 0.154 | 0.60 (0.30-1.21) |
|  | Night sleep duration | 0.002 |  | <0.001 |  | 0.266 |  |
|  | Night sleep duration (6~8 h) | 0.001 | 0.74 (0.62-0.88) | <0.001 | 0.67 (0.56-0.80) | 0.414 | 0.84 (0.55-1.28) |
|  | Night sleep duration (>8 h) | 0.334 | 0.87 (0.66-1.15) | 0.702 | 0.95 (0.72-1.25) | 0.115 | 0.46 (0.18-1.21) |
|  | CES-D (≥10) | <0.001 | 1.43 (1.22-1.69) | <0.001 | 1.40 (1.18-1.66) | 0.684 | 1.09 (0.72-1.65) |
|  | Chronic diseases (≥1) | 0.808 | 0.98 (0.80-1.19) | 0.862 | 1.02 (0.83-1.25) | 0.667 | 0.90 (0.56-1.45) |
|  | History of falls (no) | <0.001 | 0.33 (0.27-0.39) | <0.001 | 0.35 (0.29-0.42) | <0.001 | 0.24 (0.16-0.38) |
|  | Visuospatial ability (pass) | 0.379 | 0.93 (0.79-1.09) | 0.947 | 0.99 (0.84-1.18) | 0.776 | 0.94 (0.63-1.42) |

**Supplementary Table 5 |** Logistic regression analysis in subgroups divided according to education level.

| **Model** | **Variables** | **Primary school or below** | | **Middle school** | | **High school or above** | |
| --- | --- | --- | --- | --- | --- | --- | --- |
|  |  | ***P*** | **95%CI** | ***P*** | **95%CI** | ***P*** | **95%CI** |
| Model 1 | Gender (female) | 0.006 | 1.01 (1.00-1.02) | 0.012 | 1.77 (1.14-2.76) | 0.037 | 2.06 (1.05-4.04) |
|  | Age | 0.003 | 1.01 (1.00-1.02) | 0.368 | 1.01 (0.99-1.02) | 0.502 | 1.01 (0.98-1.03) |
|  | Marital status (other) | 0.198 | 1.14 (0.94-1.38) | 0.099 | 1.48 (0.93-2.34) | 0.833 | 1.08 (0.52-2.24) |
|  | Smoking (no) | 0.834 | 1.02 (0.83-1.26) | 0.611 | 0.90 (0.59-1.37) | 0.077 | 0.57 (0.31-1.06) |
|  | Physical disabilities (no) | <0.001 | 0.55 (0.43-0.71) | 0.09 | 0.59 (0.33-1.09) | 0.082 | 0.36 (0.12-1.14) |
|  | Intellectual disability (no) | 0.009 | 0.69 (0.53-0.91) | 0.474 | 0.79 (0.41-1.52) | 0.308 | 0.59 (0.22-1.62) |
|  | Night sleep duration | 0.007 |  | 0.105 |  | 0.226 |  |
|  | Night sleep duration (6~8 h) | 0.002 | 0.76 (0.64-0.90) | 0.039 | 0.66 (0.45-0.98) | 0.826 | 1.07 (0.58-1.98) |
|  | Night sleep duration (>8 h) | 0.268 | 0.85 (0.64-1.13) | 0.265 | 0.64 (0.29-1.41) | 0.101 | 2.49 (0.84-7.41) |
|  | CES-D (≥10) | <0.001 | 1.41 (1.20-1.66) | 0.425 | 0.85 (0.57-1.27) | 0.489 | 0.81 (0.44-1.48) |
|  | Chronic diseases (≥1) | 0.972 | 1.00 (0.82-1.23) | 0.047 | 1.61 (1.01-2.57) | 0.874 | 1.05 (0.55-2.02) |
|  | History of falls (no) | <0.001 | 0.33 (0.28-0.40) | <0.001 | 0.30 (0.20-0.47) | 0.04 | 0.51 (0.27-0.97) |
|  | Cognition | 0.001 | 0.97 (0.95-0.99) | 0.265 | 0.97 (0.91-1.03) | 0.915 | 1.00 (0.91-1.09) |
| Model 2 | Gender (female) | 0.004 | 1.34 (1.1-1.64) | 0.012 | 1.77 (1.14-2.75) | 0.031 | 2.09 (1.07-4.08) |
|  | Age | 0.009 | 1.01 (1.00-1.01) | 0.323 | 1.01 (0.99-1.02) | 0.402 | 1.01 (0.99-1.04) |
|  | Marital status (other) | 0.133 | 1.16 (0.96-1.41) | 0.084 | 1.50 (0.95-2.38) | 0.847 | 1.07 (0.52-2.22) |
|  | Smoking (no) | 0.853 | 1.02 (0.83-1.26) | 0.584 | 0.89 (0.58-1.36) | 0.076 | 0.57 (0.31-1.06) |
|  | Physical disabilities (no) | <0.001 | 0.54 (0.42-0.69) | 0.097 | 0.60 (0.33-1.1) | 0.089 | 0.37 (0.12-1.16) |
|  | Intellectual disability (no) | 0.004 | 0.67 (0.51-0.88) | 0.445 | 0.78 (0.40-1.49) | 0.315 | 0.60 (0.22-1.63) |
|  | Night sleep duration | 0.004 |  | 0.099 |  | 0.256 |  |
|  | Night sleep duration (6~8 h) | 0.001 | 0.75 (0.63-0.89) | 0.037 | 0.66 (0.45-0.98) | 0.802 | 1.08 (0.58-2.01) |
|  | Night sleep duration (>8 h) | 0.308 | 0.86 (0.65-1.14) | 0.259 | 0.64 (0.29-1.40) | 0.113 | 2.43 (0.81-7.25) |
|  | CES-D (≥10) | <0.001 | 1.42 (1.21-1.67) | 0.429 | 0.85 (0.57-1.27) | 0.486 | 0.81 (0.44-1.48) |
|  | Chronic diseases (≥1) | 0.875 | 0.98 (0.81-1.20) | 0.055 | 1.58 (0.99-2.52) | 0.87 | 1.06 (0.55-2.02) |
|  | History of falls (no) | <0.001 | 0.33 (0.28-0.39) | <0.001 | 0.30 (0.19-0.46) | 0.045 | 0.52 (0.27-0.99) |
|  | Orientation | 0.02 | 0.94 (0.89-0.99) | 0.193 | 0.89 (0.75-1.06) | 0.657 | 0.93 (0.68-1.28) |
| Model 3 | Gender (female) | 0.001 | 1.4 (1.15-1.71) | 0.012 | 1.77 (1.14-2.77) | 0.044 | 2.01 (1.02-3.95) |
|  | Age | 0.006 | 1.01 (1.00-1.01) | 0.563 | 1.00 (0.99-1.02) | 0.53 | 1.01 (0.99-1.03) |
|  | Marital status (other) | 0.222 | 1.13 (0.93-1.37) | 0.089 | 1.49 (0.94-2.37) | 0.818 | 1.09 (0.53-2.25) |
|  | Smoking (no) | 0.847 | 1.02 (0.83-1.26) | 0.627 | 0.90 (0.59-1.38) | 0.08 | 0.57 (0.31-1.07) |
|  | Physical disabilities (no) | <0.001 | 0.54 (0.42-0.70) | 0.089 | 0.59 (0.32-1.08) | 0.072 | 0.36 (0.12-1.10) |
|  | Intellectual disability (no) | 0.008 | 0.69 (0.53-0.91) | 0.397 | 0.76 (0.39-1.45) | 0.285 | 0.58 (0.22-1.57) |
|  | Night sleep duration | 0.005 |  | 0.092 |  | 0.217 |  |
|  | Night sleep duration (6~8 h) | 0.001 | 0.75 (0.63-0.89) | 0.032 | 0.65 (0.44-0.96) | 0.835 | 1.07 (0.58-1.98) |
|  | Night sleep duration (>8 h) | 0.255 | 0.85 (0.64-1.13) | 0.292 | 0.66 (0.30-1.44) | 0.099 | 2.50 (0.84-7.44) |
|  | CES-D (≥10) | <0.001 | 1.42 (1.20-1.67) | 0.45 | 0.86 (0.58-1.28) | 0.506 | 0.81 (0.44-1.50) |
|  | Chronic diseases (≥1) | 0.998 | 1.00 (0.82-1.22) | 0.051 | 1.59 (1.00-2.54) | 0.904 | 1.04 (0.54-2.00) |
|  | History of falls (no) | <0.001 | 0.33 (0.28-0.39) | <0.001 | 0.30 (0.20-0.46) | 0.036 | 0.51 (0.27-0.96) |
|  | Memory | <0.001 | 0.91 (0.87-0.96) | 0.367 | 0.95 (0.85-1.06) | 0.816 | 1.02 (0.87-1.19) |
| Model 4 | Gender (female) | 0.004 | 1.35 (1.10-1.65) | 0.016 | 1.72 (1.10-2.66) | 0.033 | 2.06 (1.06-4.00) |
|  | Age | 0.02 | 1.01 (1.00-1.01) | 0.519 | 1.01 (0.99-1.02) | 0.42 | 1.01 (0.99-1.03) |
|  | Marital status (other) | 0.119 | 1.17 (0.96-1.42) | 0.098 | 1.48 (0.93-2.35) | 0.848 | 1.07 (0.52-2.22) |
|  | Smoking (no) | 0.92 | 1.01 (0.82-1.25) | 0.595 | 0.89 (0.58-1.36) | 0.076 | 0.57 (0.31-1.06) |
|  | Physical disabilities (no) | <0.001 | 0.53 (0.42-0.69) | 0.08 | 0.58 (0.32-1.07) | 0.083 | 0.37 (0.12-1.14) |
|  | Intellectual disability (no) | 0.003 | 0.67 (0.51-0.88) | 0.367 | 0.74 (0.39-1.42) | 0.325 | 0.6 (0.22-1.65) |
|  | Night sleep duration | 0.004 |  | 0.081 |  | 0.248 |  |
|  | Night sleep duration (6~8 h) | 0.001 | 0.75 (0.63-0.89) | 0.027 | 0.65 (0.44-0.95) | 0.817 | 1.08 (0.58-1.99) |
|  | Night sleep duration (>8 h) | 0.335 | 0.87 (0.66-1.15) | 0.279 | 0.65 (0.29-1.42) | 0.111 | 2.44 (0.82-7.28) |
|  | CES-D (≥10) | <0.001 | 1.43 (1.21-1.68) | 0.442 | 0.86 (0.58-1.27) | 0.488 | 0.81 (0.44-1.48) |
|  | Chronic diseases (≥1) | 0.87 | 0.98 (0.81-1.20) | 0.048 | 1.60 (1.00-2.56) | 0.873 | 1.05 (0.55-2.02) |
|  | History of falls (no) | <0.001 | 0.33 (0.28-0.39) | <0.001 | 0.30 (0.19-0.46) | 0.044 | 0.52 (0.27-0.98) |
|  | Calculation | 0.103 | 0.96 (0.91-1.01) | 0.49 | 0.95 (0.81-1.10) | 0.709 | 0.96 (0.77-1.19) |
| Model 5 | Gender (female) | 0.002 | 1.37 (1.12-1.67) | 0.017 | 1.71 (1.10-2.65) | 0.037 | 2.03 (1.05-3.94) |
|  | Age | 0.04 | 1.01 (1.00-1.01) | 0.912 | 1.00 (0.99-1.02) | 0.554 | 1.01 (0.98-1.03) |
|  | Marital status (other) | 0.114 | 1.17 (0.96-1.42) | 0.078 | 1.52 (0.95-2.41) | 0.826 | 1.09 (0.53-2.24) |
|  | Smoking (no) | 0.951 | 1.01 (0.82-1.24) | 0.594 | 0.89 (0.58-1.36) | 0.086 | 0.58 (0.31-1.08) |
|  | Physical disabilities (no) | <0.001 | 0.53 (0.41-0.68) | 0.08 | 0.58 (0.32-1.07) | 0.07 | 0.35 (0.11-1.09) |
|  | Intellectual disability (no) | 0.003 | 0.66 (0.50-0.86) | 0.234 | 0.67 (0.35-1.29) | 0.281 | 0.58 (0.21-1.57) |
|  | Night sleep duration | 0.002 |  | 0.058 |  | 0.209 |  |
|  | Night sleep duration (6~8 h) | 0.001 | 0.74 (0.62-0.88) | 0.018 | 0.63 (0.42-0.92) | 0.848 | 1.06 (0.57-1.97) |
|  | Night sleep duration (>8 h) | 0.334 | 0.87 (0.66-1.15) | 0.322 | 0.67 (0.31-1.47) | 0.095 | 2.53 (0.85-7.54) |
|  | CES-D (≥10) | <0.001 | 1.43 (1.22-1.69) | 0.469 | 0.86 (0.58-1.29) | 0.499 | 0.81 (0.44-1.49) |
|  | Chronic diseases (≥1) | 0.808 | 0.98 (0.80-1.19) | 0.068 | 1.54 (0.97-2.46) | 0.889 | 1.05 (0.55-2.00) |
|  | History of falls (no) | <0.001 | 0.33 (0.27-0.39) | <0.001 | 0.29 (0.19-0.44) | 0.036 | 0.51 (0.27-0.96) |
|  | Visuospatial ability (pass) | 0.379 | 0.93 (0.79-1.09) | 0.279 | 1.31 (0.80-2.13) | 0.763 | 1.14 (0.49-2.61) |
